# Anti-inflammatory, anti-oxidant, and immunomodulatory activities of the genus *Ferula *and their constituents: A review

**DOI:** 10.22038/IJBMS.2021.59473.13204

**Published:** 2021-12

**Authors:** Zahra Ghasemi, Ramin Rezaee, Mohammad Reza Aslani, Mohammad Hossein Boskabady

**Affiliations:** 1 Applied Biomedical Research Center, Mashhad University of Medical Sciences, Mashhad, Iran; 2 Department of Physiology, Faculty of Medicine, Mashhad University of Medical Sciences, Mashhad, Iran; 3 Clinical Research Unit, Imam Reza Hospital, Faculty of Medicine, Mashhad University of Medical Sciences, Mashhad, Iran; 4 Department of Physiology, Faculty of Medicine, Ardabil University of Medical Sciences, Ardabil, Iran

**Keywords:** Anti-inflammatory, Anti-oxidant, Coumarins, Ferula, Immonumodulatory

## Abstract

*Ferula* is a genus of the family *Apiaceae* and it includes around 170 species of flowering plants mostly native to the Mediterranean region and eastern to central Asia. In Iran, *Ferula* spp. are widely used in cuisine and traditional medicine. This review discusses the anti-inflammatory, anti-oxidant, and immunomodulatory activities of different species of *Ferula*. To prepare the present review, Scopus, Google Scholar, PubMed, and Web of Science scientific databases were searched to retrieve relevant articles published from 1985 until December 2020. Based on our literature review, *Ferula *plants and their derivatives decrease the levels of inflammatory mediators and exert anti-apoptotic effects. Under oxidative stress conditions, these plants and their constituents were shown to decrease oxidative markers such as malondialdehyde, reactive oxygen species, and nitric oxide but increase superoxide dismutase, glutathione peroxidase, catalase activity, and glutathione level. *Ferula *plants and their constituents also showed immunomodulatory effects by affecting various cytokines. Besides, *in vivo* and *in vitro* studies showed hypotensive, neuroprotective, memory-enhancing, anti-oxidant, hepatoprotective, antimicrobial, anticarcinogenic, anticytotoxic, antiobesity, and anthelmintic effects for various species of *Ferula *and their constituents. These plants also showed a healing effect on gynecological issues such as miscarriage, unusual pain, difficult menstruation, and leukorrhea. All these beneficial effects could have resulted from the anti-inflammatory, anti-oxidant, and immunomodulatory effects of these plants and their constituents. Based on the available literature, members of the genus *Ferula *can be regarded as potential therapeutics against inflammatory conditions, oxidative stress, and immune dysregulation.

## Introduction

For centuries, herbs have been used for treatment of different diseases (1). During the past decades, assessment of beneficial health effects of these herbal plants has introduced them as invaluable sources of active compounds that may potentially serve as drugs. Members of the genus *Ferula* have been traditionally used as anti-oxidant, anticancer, carminative, antinociceptive, antibacterial, antiviral, aphrodisiac, expectorant, and diuretic agents to treat neurological conditions, dizziness, headache, rheumatism, inflammation, bronchitis, asthma, and gastrointestinal disorders (2). Considering the beneficial effects of *Ferula* species on respiratory conditions, the relaxant effect of these plants and their constituents on tracheal smooth muscles (3, 4) and their inhibitory effect on muscarinic receptors were reported (5-7).

In various parts of the world, different species of *Ferula* have been used in traditional medicine. In Afghanistan, dried gum of *F. assafoetida* soaked in warm water has been used for treatment of ulcers, whooping cough, and anxiety. In Morocco, *F. communis* is used as an antispasmodic agent and *F. assafoetida* as an anti-epileptic remedy. In Nepal, an aqueous extract of* F. assafoetida* is used orally as an anthelmintic agent. In addition, in Saudi Arabia, these plants are used for treatment of asthma, bronchitis, and cough. Leaves and stems of *F. assafoetida* are also used to treat erectile dysfunction. In the USA and by black American people, resin extract of *F. assafoetida* is used against cancer, menstrual problems, asthma, convulsion, and laryngitis and is known as an antispasmodic agent (8). *F. assafoetida* is also used for the treatment of functional dyspepsia, bloating, postprandial fullness, and digestive problems (9). Traditionally, *F. assafoetida* has been used for prevention of abortion and treatment of painful menstruation and leukorrhea. *F. assafoetida* has also been reported to be effective in the treatment of gastric diseases through increasing saliva secretion and inducing amylase activity. These plants were shown to suppress gastric acid secretion and gastric pressure, increase high-fat digestion through bile acid secretion, and facilitate defecation (10).

Therapeutic effects of *Ferula* plants are mediated via different mechanisms such as induction of apoptosis, inhibition of lipoxygenase, cyclooxygenase (COX), and inducible nitric oxide synthase (iNOS), reduction of nitric oxide (NO) and prostaglandin E2 (PGE2) levels, modulation of heat shock protein 70 (Hsp70), and reduction of tumor necrosis factor (TNF)-α and interleukin (IL)-6 (11).

*F. assafoetida *gum and its root gum resin showed hypoglycemic, hypolipidemic, and gastroprotective effects. In India, it is also used for the treatment of abdominal bloating, flatulence, and gastric disorders. Recent pharmacological studies showed anti-oxidant, anti-diabetic, laxative, anticancer, antiviral, and antifungal effects of this plant (12). *Ferula longipes* Coss has anti-tumor and anti-inflammatory activities and contains sesquiterpene chromone derivatives, daucane esters, and prenyl-benzoyl-furanone-type sesquiterpenoids (13). Some *Ferula* species from Iran and Russia have shown estrogen-like activity, with significant cytotoxicity against MCF-7, HepG2, and MDBK cell lines (14). 

The present review provides a summary of anti-inflammatory, anti-oxidant, and immunomodulatory properties of *Ferula* members shown by experimental and clinical studies. 

## Methods

To prepare the present review, scientific databases Scopus, Google Scholar, PubMed, and Web of Science were searched using keywords such as *Ferula*, inflammation, anti-oxidant, and immunomodulatory to retrieve relevant articles published from 1985 until December 2020.

***Different plants and their chemical composition***

Phytochemical screening of the genus *Ferula* (*F.*) revealed more than 200 chemical structures including sesquiterpene coumarins and coumarin esters. The molecular structure of the major chemical compounds found in various extracts/oils from different *Ferula* plants is shown in Figures 1 and 2. 

Umbelliprenin and farnesiferols A and B are three important compounds present in *F. persica*. The odor and taste of *F. persica* are due to the presence of sulfur and persicasulfide A, B, and C which are the major sulfur compounds present in *F. persica* (15). *F. diversivittata*, another species of *Ferula*, contains compounds such as umbelliprenin (UMB) and auraptene which showed various pharmacological activities (16-20). The Prenyl chain of diversin has main roles in its antigenotoxic and anti-tumor properties (21). *Ferula *species possess a strong aromatic smell that is due to the presence of oleoresin (22). 

***Anti-inflammatory activities of Ferula plants***

The anti-inflammatory and anti-nociceptive effects of *F. assafoetida* (2.5, 5, 10, and 20 mg/kg) were evaluated in mice; results showed a significant anti-nociceptive effect especially at 10 mg/kg. In addition, this study evaluated the anti-inflammatory effects of the plant on carrageenan-induced mice paw edema and results revealed that paw weight was significantly reduced at 2.5 mg/kg *F. assafoetida*, suggesting anti-inflammatory and analgesic effects of the plant (23). In another study, Bagheri *et al*. also examined the antinociceptive effect of *F. assafoetida* seed essential oil at doses of 2.5, 5, and 10 mg/kg and compared it with that of morphine sulfate or sodium diclofenac in mice. The results showed an antinociceptive effect for the oil on chronic and acute pain and it was concluded that this effect might be produced through an anti-inflammatory function or by modulating the central opioid pathways (24). The cytotoxic activity of *F. assafoetida* has also been reported in some studies. The oleo-gum-resin of *F. assafoetida*, as well as methanol extracts of different *Ferula* species, showed dose-dependent cytotoxic effects (25). Also, the cytotoxic function of gum resin of *F. assafoetida* on senescent fibroblasts, at concentrations above 5×10^-7^ g/ml, led to cell death, while at concentrations between 5×10^-8^ and 10^-7^ g/ml it showed revitalizing effects (26).

Hydroalcoholic extract of *F. szowitsiana* DC (50, 100, 200, and 400 mg/kg, intraperitoneal (IP)) dose-dependently reduced inflammation induced by formalin in Wistar rats (27). Askari *et al*. also showed that the methanolic extract of *F. szowitsiana* root (10–160 mg/ml) significantly reduced inflammatory cytokines (interleukin (IL)-6 and tumor necrosis factor (TNF)-α) in phytohemagglutinin (PHA)-stimulated isolated human lymphocytes, indicating its anti-inflammatory effects (28).

The effects of aqueous, methanolic, and acetone extracts of the seed and root of *F. gummosa *Boiss on acute and chronic pain, as well as inflammation, were examined. Only the acetone extract of the root could reduce licking and biting time in the late phase of the formalin test (used for chronic pain assessment) but the extracts did not show anti-inflammatory effects (29). In an *in vitro* study, the antibacterial and anti-inflammatory effect of *F. hermonis* was observed at a concentration of 25 μg/ml (30). 

***Anti-inflammatory activities of Ferula constituents***

A combination of traditional Chinese medicine “Awei” containing six bioactive sesquiterpene coumarins from *F. sinkiangen* extract was examined for possible anti-inflammatory effects in BV-2 microglial cells. The anti-neuroinflammatory activities of Awei were revealed by lower mRNA expression of inflammatory cytokines IL-6, TNF-α, and IL1β (31).

An *in vitro* study showed that the two main compounds of *Ferula*, UMB and methyl galbanate (MG) have anti-inflammatory effects (32). A study by Zamani *et al*. showed that phytohemagglutinin (PHA) stimulated splenocyte proliferation was significantly reduced in the presence of UMB and MG (32). In an *in vivo* study, the anti-inflammatory effect of UMB in carrageenan-induced paw edema was revealed (33). Also, UMB derived from *F. szowitsiana* demonstrated cytotoxic and cytostatic effects in human solid cancer cells (metastatic pigmented malignant melanoma (M4Beu)); UMB at 25 µM reduced serum-induced proliferation of M4Beu through cell cycle blockade in G1 and induction of apoptosis (34).

The anti-inflammatory effects of three main compounds from *F. hermonis* root oil namely, ferutinin, teferin, and eferidin on carrageenan-induced edema were evaluated in rats. Both ferutinin and teferin showed anti-inflammatory effects at a dose of 100 mg/kg, while teferidin showed no anti-inflammatory activity (35). It was also shown that ferutinin is an agonist for the estrogen receptor (ER)-α and an agonist/antagonist for ERβ with minor anti-proliferative properties in breast cancer cells. Through esterification of jaeschkenadiol with different acids, ferutinin analogues were synthesized to increase its anti-proliferative activity. *In vitro*, ferutinin analogues exerted anti-proliferative activities in both estrogen-dependent and estrogen-independent cell lines of breast cancer (36).

The anti-inflammatory activities of coumarin (1,2-benzopyrone) and warfarin (4-hydroxycoumarin) which are other constituents of *Ferula* were also reported. In diseases such as post-mastectomy lymphoedema, it has been shown that coumarin(s) reduced inflammatory processes by macrophage-induced proteolysis of edema protein, lowering protein levels, and reducing their binding and preventing their filtration from capillary pores to tissues as well as inhibiting the pro-inflammatory 5-lipoxygenase enzyme (37).

In a study, anti-inflammatory effects of auraptene were compared with UMB in a mouse skin model using 12O-tetradecanoyl-phorbol-13-acetate; pretreatment of the skin with auraptene significantly suppressed leukocyte infiltration, edema formation, cell proliferation, and hydrogen peroxide production, which were not seen in the UMB-pretreated group (38). The inhibitory effects of auraptene and UMB against promastigotes of *Leishmania* major were also demonstrated *in vitro*. The results showed significant activity of auraptene and UMB at IC_50_ (5.1 µg/ml and 4.9 µg/ml, respectively) (39). Also, auraptene reduced edema by 50% in croton oil-induced edematous response, in an animal model of acute inflammation (40). Auraptene exerted anti-inflammatory effects in nonalcoholic fatty liver disease (NAFLD) as it decreased TNF-α and triglycerides but increased adiponectin and peroxisome proliferator-activated receptors-α (PPARα) (41).

As stated above, *Ferula* plants and their constitutes exert anti-inflammatory effects. *F. assafoetida* showed anti-nociceptive properties against chronic and acute pain and carrageenan-induced mice paw edema. *F. szowitsiana *reduced formalin-induced inflammation in rats and downregulated IL-6 and TNF-α in PHA-stimulated human lymphocytes. *F. gummosa* reduced chronic pain and *F. hermonis* also showed anti-inflammatory properties. 

The anti-inflammatory effects of bioactive sesquiterpene coumarins, as well as UMB and MG, were shown. For instance, UMB inhibited carrageenan-induced paw edema. The anti-inflammatory effects of ferutinin, teferin, and teferidin on carrageenan-induced edema were shown. Auraptene also showed anti-inflammatory effects as it suppressed leukocyte infiltration, edema formation, cell proliferation, and hydrogen peroxide production, and inhibited croton oil-induced edematous response by decreasing TNF-α and triglycerides but increasing adiponectin and PPARα. A summary of the anti-inflammatory effects of *Ferula* species and their constituents is shown in Tables 1 and 2. 

***Anti-oxidant effects***

The imbalance between production of oxidants (free radicals) and the anti-oxidant defense system is called oxidative stress. The accumulation of oxidized lipid plays an important role in a variety of diseases such as cardiovascular, lung, gastrointestinal, and kidney diseases as well as diabetes, cancer, and aging. Therefore, identifying compounds that reduce or prevent the production of oxidant products can be useful in the treatment of these diseases (42). Various molecules including reactive oxygen species (ROS), reactive nitrogen species (RNS), hydrogen peroxide (H_2_O_2_), and thiobarbituric acid reactive substances (TBARS) are involved in the oxidative stress process (43) and they damage DNA and major proteins. Under physiological conditions, anti-oxidant factors protect cells against destruction by oxidative molecules (44).

The anti-oxidant system is a set of enzymes such as superoxide dismutase (SOD) and catalase (CAT), biological macromolecule structures such as albumin, ceruloplasmin, ferritin and other small molecules, molecules such as ascorbic acid, alpha-tocopherol, carotenoids, polyphenols, ubiquinol-10, reduced glutathione (GSH), methionine, uric acid, and bilirubin (45). The effects of *Ferula* plants on oxidative stress are discussed below. 

***Anti-oxidant activities of Ferula plants***

The methanolic extract of *F. szowitsiana* root decreased malondialdehyde (MDA), ROS, NO, IL-6, and TNF-α levels but increased SOD and GSH levels which confirmed the anti-inflammatory and anti-oxidant effects of the plant (46). The anti-diabetic and anti-oxidant effects of the methanol extracts of *F. drudeana* Korovin and *F. huber-morathii* Peşmen in streptozotocin (STZ) -induced diabetic rats were investigated; it was found that after 14 and 28 days of oral treatment with *F. drudeana* (400 mg/kg) and *F. huber-morathii* (200 and 400 mg/kg) extracts, plasma levels of fasting blood glucose, glycosylated hemoglobin (HbA1c), triglycerides (TG), total cholesterol (TC), low-density lipoprotein (LDL) were significantly reduced, while insulin levels were significantly increased. Both extracts also significantly increased the activity of SOD, glutathione peroxidase (GPx), and CAT and GSH levels in homogenized liver and pancreas tissues of diabetic rats. In addition, both extracts improved alanine transaminase (ALT), aspartate transaminase (AST), alkaline phosphatase (ALP), bilirubin, total protein, high-density lipoprotein (HDL) serum levels, and MDA in homogenized liver and pancreas tissues in the treated diabetic groups (47). Ethanol extract of *F. assafoetida* and its essential oil were examined for anti-oxidant activities. The IC_50_ values for NO-scavenging activity and Fe^2^ chelating ability of the extracts were 270±3 and 0.57±0.02 mg/ml, respectively. The results also showed that the anti-oxidant activity of the extracts (peroxidation inhibition) in the interval of 24 to 72 hr was 82–88% (48). The anti-oxidant and anti-carcinogenic effects of *F. assafoetida* on 12-o-tetradecanoyl 13-phorbol acetate (TPA)-mediated cutaneous oxidative stress in Swiss albino mice, have also been investigated. Pretreatment with *F. assafoetida* (300, 400, and 500 µg/200 µl acetone/animal), increased the contents of hydrogen peroxide and xanthine oxidase activities, and carbonyl protein in the skin of mice (49). The anti-oxidant effects of different concentrations of hydroalcoholic extract of *F. foetida* (200, 400, and 800 mg/kg, orally) were shown in rats. On the other hand, although hydroalcoholic extract of *F. foetida* stems did not affect plasma H_2_O_2_ content, it resulted in a significant increase in ferric reducing anti-oxidant power (50). Treatment of rats with gentamicin-induced renal impairment with *F. foetida* extract, improved the levels of blood urea nitrogen (BUN), serum creatinine, and TBARS (51). The chemopreventive and anti-oxidant effects of *F. assafoetida* (1.25 and 2.5% w/w in diet) on N-methyl-N-nitrosourea (MNU)-induced mammary carcinogenesis in Sprague-Dawley rats were shown by increased activities of glutathione S-transferase, DT-diaphorase, SOD, CAT, and GSH level and decreased cytochrome P450 and b5. Treatment with *F. assafoetida* also modified the anti-oxidant system so that lipid peroxidation was significantly inhibited in rats’ liver tissue (52). The anti-oxidant and chemotherapeutic effects of *F. assafoetida* hydroalcoholic extract (6.25 and 12.5 mg/Kg, b.w), were also reported in 1,2-dimethyl hydrazine-induced colon carcinogenesis in Wistar rats; the extract reduced the levels of cytochrome P_450_, β-catenin, and ferric reducing ability of plasma. The levels of GSH and glutathione S-transferase were also significantly increased in the *F. assafoetida* extract-treated animals (53). In carps, effects of different doses (0, 0.5, 1, and 2%) of *F. assafoetida* given for 8 weeks were examined on the expression of anti-oxidant enzymes Glutathione reductase (GSR), GPx, and Glutathione S-Transferase Alpha (GSTA) as well as growth genes growth factor *(GH*), *IGF1* (insulin growth factor 1) and Ghrelin and Obestatin Prepropeptide *(Ghrl*); treatments significantly increased GSR and GSTA anti-oxidant factors in a dose-dependent manner and significantly enhanced the expression of growth-related genes (54).

Anti-oxidant and antitumor effects of four different fractions of *F. sinkiangensis* K. M. Shen (petroleum ether, ethyl acetate, n-butanol, and methanol) were demonstrated in HCT116, Caco-2, HepG2, and MFC cells. Petroleum ether fraction showed significant anti-oxidant effects at all concentrations, while ethyl acetate, n-butanol, and methanol fractions showed free radical-scavenging activities in a dose-dependent way. *Ferula* fractions also inhibited proliferation and increased apoptosis dose-dependently (55).

The effects of daily oral administration of *F. gummosa* root hydroalcoholic extract (100 and 600 mg/kg) on oxidant-anti-oxidant status were reported in an *in vivo* study; the results showed that 28-day administration of both concentrations of the extracts increased the activities of CAT and SOD enzymes, which was more pronounced at 600 mg/kg. On the other hand, *F. gummosa* extract decreased serum MDA levels but did not affect total thiol serum levels (56).

The hepatoprotective effects of *F. communis* extract on tetrachloride (CCl_4_)-induced oxidative damage were studied in rats; eight-week administration of the extract (150 and 300 mg/kg) caused a decrease in serum levels of AST, ALT, γglutamyl transferase (GGT), and total bilirubin (T-BIL), while activities of anti-oxidant enzymes SOD and GPx increased in the liver, which were more pronounced at 150 mg/kg. The results showed that *F. communis* extract was effective against oxidative damage induced by CCl_4_ (57).

The anti-oxidant activities of kamolonol acetate extracted from* F. pseudalliacea* have been reported *in vitro* against colorectal cancer cell lines (i.e., HCT116 and CT26). The results showed a strong anti-oxidant activity for kamolonol acetate (58). Another study showed the anti-oxidant effects of 17 daucane sesquiterpenoid esters isolated from *F. hermonis* (59). Daucane sesquiterpenoid esters showed anti-oxidant effects and inhibited 1,1-diphenyl-2- picryl hydrazyl (DPPH) oxidation and production of thiobarbituric acid reactive substances (TBARS) (30). Miski *et al*. also showed the anti-oxidant effects of daucane esters extracted from *F. rigidula* (60). Moderate anti-oxidant activity of essential oils extracted from the leaves of *F. vesceritensis* Coss. et Dur. has been reported (61). The essential oil of *F. heuffelii* also showed anti-oxidant activity (62). In an *in vitro* study, flower- and leaves-derived oil of *F. tingitana* showed considerable cytotoxic effects against breast (MCF7), cervical (HELA), and liver (HEPG2) carcinoma cell lines (63). The anti-oxidant effects of various extracts (chloroform, ethyl acetate, and methanol) from the aerial parts of *F. caspica* M. Bieb were assessed and results revealed that chloroform and ethyl acetate extracts had the highest anti-oxidant effects (64).

In a clinical trial study on 75 children with leukemia, the anti-oxidant effect and metabolic regulatory properties of *F. assafoetida* extract (50 and 100 mg, daily for 45 days) were investigated; in children receiving the extract, fasting blood sugar, and plasma levels of TC, TG, and LDL were significantly reduced compared with the placebo group, while plasma levels of HDL were elevated. In addition, the extract significantly increased SOD, CAT, and total anti-oxidant capacity in the treated group compared with the placebo group, while the plasma levels of MDA and protein carbonyl were decreased (65). 

***Anti-oxidant activities of Ferula constituents***

The anti-oxidant effects of auraptene, one of the main constituents of *Ferula* species were shown in several studies. Auraptene from *F. szowitsiana* showed antigenotoxic effects on DNA damage in human T cells possibly through suppression of superoxide anion (O2-) generation; auraptene (5, 10, 25, 50, 100, 200, and 400 mM) significantly reduced the genotoxicity induced by H_2_O_2_ and this effect was comparable to or even better than that of ascorbic acid (66). The anti-oxidant effects of auraptene (12.5, 25, and 50 mg/kg) have also been investigated in the brain tissue of kindling mice induced by repeated IP injections of pentylenetetrazol. Although auraptene had no significant effect on MDA concentrations in the brain tissue, at 50 mg/kg, it increased GSH level (67). Ghanbarabadi *et al*. also examined the anti-oxidant effects of auraptene (4, 8, and 25 mg/kg, orally) in a standard animal model of vascular dementia and chronic cerebral hypoperfusion; the results showed that auraptene decreased MDA but increased GSH content in the cortex and hippocampus tissues (68). The anti-oxidant effects of auraptene in NAFLD were also reported (41). Anti-inflammatory and anti-oxidative effects of auraptene were widely reported (69, 70).

In lymphocytes with DNA damage induced by H_2_O_2_, UMB (10, 25, 50, 100, 200, and 400 μM) exerted protective effects in a dose-dependent manner (71). In a study, the effect of five sesquiterpene chromone derivatives, fukanefurochromones AE (1-5), on the production of NO and inducible NO synthase (iNOS) gene expression was tested in a murine macrophage-like cell line (RAW 264.7) activated by lipopolysaccharide (LPS) and recombinant mouse interferon-γ (IFN-γ); the results showed that sesquiterpene chromone derivatives significantly inhibited NO production and iNOS gene expression (72).

As noted in this section, several studies showed anti-oxidant properties of* Ferula* plants and their constitutes. *F. szowitsiana* root extract showed anti-oxidant effects and decreased MDA, ROS, and NO but increased SOD levels. *F. drudeana* and *F. huber-morathii* also reduced MDA levels but increased SOD, GPx, and CAT activities and GSH levels in homogenized liver and pancreas tissues. *F. assafoetida* showed nitric oxide-scavenging activity and Fe^2^ chelating ability, increased hydrogen peroxide and xanthine oxidase activities, and carbonyl protein and increased ferric reducing anti-oxidant power and glutathione S-transferase activity. DT-diaphorase, SOD, and CAT, activities while GSH level was decreased and lipid peroxidation was inhibited, expression of anti-oxidant enzymes (GSR, GPx and GSTA) and growth genes (*GH*, *IGF1*, and *Ghrl*) were increased by *F. assafoetida*. The anti-oxidant effects of fractions of *F. sinkiangensis* and *F. gummosa* were also demonstrated by increased activities of CAT and SOD but decreased MDA serum levels. *P. ferulae* has shown anti-oxidant activity via β-carotene-linoleic acid. These compounds have chelating and scavenging properties. *F. communis* extract decreased GGT serum level but increased anti-oxidant activities of SOD and GPx in the liver with oxidative damage and *F. pseudalliacea* showed DPPH radical scavenging. The anti-oxidant effects of 17 daucane sesquiterpenoid esters were shown by inhibiting radical scavenging activity and decreasing products of lipid peroxidation such as DPPH and TBARS. *F. rigidula*, *F. heuffelii*, and *F. vesceritensis* also showed DPPH radical scavenging and TBARS assays. The anti-oxidant effects of extracts of *F. caspica* and *F. tingitana* were also reported.

Auraptene showed antigenotoxic effects on DNA damage through increasing SOD and GSH levels but decreasing MDA. Sesquiterpene coumarins, farnesiferol A, and galbanic acid increased intracellular ROS. UMB showed a dose-dependent protective activity in lymphocytes with H_2_O_2_-induced DNA damage and five sesquiterpene chromone derivatives, fukanefurochromones A-E, inhibited NO production and iNOS gene expression. Anti-oxidant effects of *Ferula* species and their constituents are summarized in Tables 3 and 4. 

***Immunomodulatory effects***

The main task of the immune system is to defend against pathogens. The first line of defense in this system are lymphocytes, neutrophils, and monocytes/macrophages which are known as phagocytes. Other functions of phagocytic cells are chemotaxis and degradation of biological pathogens. Some molecules derived from medicinal plants alter the immunomodulatory activity of these phagocytic cells. Essential oils derived from these plants can increase or decrease the activity of the immune system based on their chemical composition. Some of such compounds are terpenes and several other natural agents present in essential oils of *Ferula* species which have shown immunomodulatory properties (73). 

***Immunomodulatory activities of Ferula plants***

In a study, gene expression of *TNF-α*, *IL-1β*, *IL-8*, and lysozyme (LYZ) was assessed in carp after 8 weeks of feeding with different levels (0, 0.5, 1, and 2%) of *F. assafoetida*; results showed increased expression of the examined genes (54).

*F. iliensis* essential oils stimulated [Ca^2+^]i mobilization in human neutrophils and activated ROS production in human neutrophils and murine bone marrow phagocytes which were dose-dependently inhibited by capsazepine, a TRPV1 channel antagonist. The essential oils also stimulated Ca^2+^ influx in TRPV1 channel–transfected HEK293 cells and desensitized the capsaicin-induced response (73).

*F. szowitsiana* methanolic extract (10, 40, and 160 µg/ml) concentration-dependently inhibited cell proliferation, and reduced IFN-γ, IL-4, and IL-10 secretion as well as their gene expression and NO production but increased IFN-γ/IL-4 and IL-10/IL-4 ratios (T helper 1/Th2 and Treg/Th2 balances, respectively) in human lymphocytes stimulated by LPS. These findings suggest the possible therapeutic effect of the plant’s extract in inflammatory diseases with dominant Th2 activity (74). 

The plants and constituents of the *Ferula* genus also revealed different immunomodulatory effects. It was shown that *F. assafoetida*, increased TNF-α, IL-1β, IL-8, and lysozyme (LYZ) in serum and growth of factors such as GH, IGF1, and Ghrl. *F. szowitsiana* extract showed an inhibitory effect on cytokines secretion, NO production, and genes expression but increased IFN-γ/IL-4 and IL-10/IL-4 ratios (T helper 1/Th2 and Treg/Th2 balances, respectively). 

***Immunomodulatory activities of Ferula constituents ***

An *in vitro* study showed the immunomodulatory properties of UMB and MG on immune cells isolated from naive mice. The results indicated that both compounds induced IL-4 but suppressed IFN- γ secretion. In addition, both UMB and MG suppressed LPS-stimulated splenocytes’ production of NO and PGE2 and significantly reduced the expression of iNOS and COX (32). The inhibitory effect of UMB on lipoxygenase was shown to potentially decrease leukotriene production (33).

The constituents of *F. szowitsiana* such as UMB, MG, and terpenoid coumarins decreased the levels of inflammatory cytokines such as IL-4 and shifted the immune system from Th1 to Th2 or CD4+/CD8+ ratio by inhibition of IL-4 but increasing INF-γ levels. The inhibitory effects of auraptene on T-cell proliferation and division were shown to be mediated at low concentrations (10 and 20 μM) by reduction of CD3/CD28-activated T lymphocyte and at high concentrations (40 μM) by decreasing serum IL-4 levels (75).

The immunomodulatory effects of auraptene in *in vitro* and *in vivo* studies were shown. In the *in vitro* study, auraptene increased IgM production in human HB4C5 cell hybridoma, stimulated IgA and IgG production in primary mouse splenocytes, and induced IgA and IgM production by lymphocytes from mesenteric lymph nodes. However, in the *in vivo* study, after 14 days of oral administration, auraptene (40 and 200 mg/kg) activated immunoglobulin production in splenocytes and lymphocytes from mesenteric lymph nodes, induced IL-4, IFN-γ, and TNF-α production in splenocytes activated by concanavalin A, and increased B cell population in splenocytes (76). The immunomodulatory effects of auraptene (10, 30, and 90 μM) on PHA-stimulated and nonstimulated human isolated lymphocytes were reported; results showed that all three concentrations of auraptene significantly reduced cell proliferation and IL-4, IL-10, IFN-γ, NF-kB, and NO levels in PHA-stimulated cells. On the other hand, although *IL-10* and *IL-4* gene expression was decreased as a result of auraptene treatment, IFN-γ expression, as well as IFN-γ/IL-4 and IL-10/IL-4 ratios, were significantly increased by all concentrations (77). The effects of auraptene on PGE2 and COX-2 in LPS-stimulated RAW 264.7 cells were examined; in this study, auraptene inhibited the production of PGE2 in LPS-stimulated macrophage cells and increased the expression of COX-2 mRNA, but significantly reduced COX-2 protein level, indicating posttranscriptional inhibitory effects of the compound (78). The effects of six terpenoid coumarins (i.e., MG, galbanic acid, farnesiferol A, badrakemone, UMB, and auraptene) extracted from *F. szowitsiana* DC. on NO production in RAW264.7 mouse macrophage cells stimulated with LPS and IFN-γ, were also examined; the results showed that among 6 terpenoids, MG significantly reduced NO production as well as iNOS mRNA expression level in LPS/IFN-γ-stimulated RAW264.7 cells. Decreased expression of COX-2 mRNA was also partially induced by MG (79).

Treatment of neutrophils with β-pinene, sabinene, γ-terpinene, geranylacetone, and isobornyl acetate, the constituents of essential oil of *F. akitschkensis*, desensitized the cells to N-formyl-Met-Leu-Phe (fMLF) and IL-8-induced [Ca^2+^]i flux and inhibited fMLF-induced chemotaxis which was inhibited by transient receptor potential (TRP) channel blockers. However, myristicin inhibited neutrophil [Ca^2+^]i flux stimulated by fMLF and IL-8 and inhibited capsaicin-induced Ca^2+^ influx in TRPV1-transfected HEK293 cells. These findings suggest that these effects of *F. akitschkensis* may be mediated via modulation of TRP channels (80).

As stated in this section, the immunomodulatory activities of *Ferula* plants and their constituent were shown in several studies. Increased IL-4 but suppressed IFN- γ secretion by UMB and MG were shown in immune cells. The effects of UMB, MG, and terpenoid coumarins on reduction of IL-4 level, increasing IFN-γ, and shifting the immune system from Th1 to Th2 or CD4+/CD8+ were reported. Auraptene increased IgM and IgG production and activated immunoglobulin production in splenocytes and lymphocytes, induced IL-4, IFN-γ, and TNF-α production in splenocytes treated with concanavalin A. In PHA-stimulated and non-stimulated human lymphocytes, auraptene reduced cytokines (IL-4, IL-10, and IFN-γ), NF-kB levels, and NO production but increased IFN-γ/IL-4 and IL-10/IL-4 ratio and inhibited TNF-α. The results showed the effects of the *Ferula* plant and its constituents improve the cellular immune system but this, in turn, could affect humoral immunity. A summary of the immunomodulatory effects of *Ferula* species and their constituents is given in Table 5. 

**Table 1:** Anti-inflammatory effects of the extract of *Ferula* species

Extract	Doses	Model of study	Effects	Ref.
*F. szowitsiana* methanolic E.	10, 40 and 160 μg/ml	PHA-stimulated human T lymphocytes	Decreased IL-6 and TNF-α levels and attenuated the overproduction of inflammatory markers	(46)
*F. assa-foetida* Oleo Gum Resin	10 and 20 mg/kg, IP injection	Carrageenan-induced mice paw edema, hot plate test	Induced anti-nociception and inhibited lipoxygenase activity	(23)
*F. * *assafoetida* seed’s essential oil of	2.5, 5, and 10 mg/kg	Acetic acid-induced writhing tests in mice, hot plate tests.	Induced anti-nociception and showed anti-inflammatory activities	(24)
*F. * *assafoetida* Methanol E.	6-321 μg/mL	PTZ-induced seizures in mice	Cytotoxic activity	(25)
*F. szowitsiana* hydroalcoholic E.	50, 100, 200, 400 mg/kg, IP injection	Formalin-induced inflammation in Wistar rats	Reduced inflammation in a dose-dependent manner, mediated pain by the opioid system	(27)
*F. gummosa *seed and root acetone E.	200, 300, 400, and 500 mg/kg, IP injection	Acute and chronic pain in mice and rat	Showed anti-inflammatory effects	(29)
*F. hermonis*	25 μg/mL	*In* * vitro* study of pathogenic bacteria (*Agrobacterium tumefaciens*, *Erwinia* sp., *Klebsiella pneumonia*, and *Pseudomonas aeruginosa*)	Showed antibacterial and anti-inflammatory effects	(30)
*F, sinkiangen *E.	0.25 mg/mL	BV-2 microglial cells	Reduce IL-6, TNF-α, and IL-1β	(31)

PHA: phytohemagglutinin, IP: intraperitoneal, STZ: Streptozotocin, IL-6: interleukin 6, TNF- α: tumor necrosis factor-alpha, E: extract

**Table 2:** Anti-inflammatory effects of the constituents of *Ferula* species

Constituents	Doses	Model of study	Effects	Ref.
Umbelliprenin	5–15 µM	PHA-stimulated splenocytes	Decreased IFN-γ and IL-4 cytokine levels	(32)
Umbelliprenin	10 μl	Carrageenin-induced rat paw edema	Showed anti-inflammatory effects and inhibited carrageenan-induced edema	(33)
Umbelliprenin	25 µM	Human solid cancer cells (melanoma= M4Beu)	Showed cytotoxic and cytostatic effects and induced apoptosis	(34)
Ferutinin and teferin	100 mg/kg	Carrageenan-induced edema model in rats	Showed anti-inflammatory effects and suppressed histamine and/or serotonin actions	(35)
Ferutinin	1-50 µM	MCF-7 and MDA-MB-231 breast cancer cells	Showed anti-proliferative properties	(36)
Auraptene	810 nmol in 100 μl acetone	RAW 264.7	Reduced leukocyte infiltration, edema formation, cell proliferation, H_2_O_2_ production, and suppressed NO synthase, PGE2, nitrite anion, and TNF-α levels.	(38)
Auraptene	IC_50_ values 5.1 µg/ml	Promastigotes of Leishmania major	Showed anti-leishmanial effects	(39)
Umbelliprenin	IC_50_ values 4.9 µg/ml	Promastigotes of Leishmania major	Showed anti-leishmanial effects	(39)
Auraptene	1.00 µmol/cm^2^	Croton oil-induced edematous response in mice	Reduced edema	(40)
Auraptene		Nonalcoholic fatty liver disease model	Decreased TNF-α and triglycerides but increased adiponectin and PPARα	(41)

PHA: phytohemagglutinin, PGE2: prostaglandin E2, NO: nitric oxide, PPARα: peroxisome proliferator-activated receptors-α, TNF-α: tumor necrosis factor-alpha, IFN-γ: interferon-gamma, IL-4: interleukin 4, MCF-7: Michigan Cancer Foundation-7, H2O2: Hydrogen peroxide

**Table 3:** Anti-oxidant effects of the extract of *Ferula* species

Extract	Doses	Model of study	Effects	Ref.
*F. gummosa* Hydroalcoholic extract	90 mg/kg/day gavage, 8 weeks	l-NAME-induced oxidative stress in rats renal tissues	Decreased lipid peroxidation, TBARS, and SOD	(11)
*F. caspica *chloroform, ethyl acetate, and methanol extracts	20 µL	Folin-Ciocalteu and aluminum chloride methods	High antioxidant effects	(64)
*F. drudeana* (F. drudeana) methanol extracts	400 mg/kg, orally	STZ-induced diabetic rat	Reduced FBS, TG, TC, LDL, and HbA1c, ALT, AST, ALP, bilirubin, HDL, MDA, increased SOD, GPx, CAT, and GSH activities, and insulin	(47)
*F. huber-morathii *(*F. Huber-morathii*) M. E.	200 and 400 mg/kg (Oral)	STZ-induced diabetic rats	Recovered levels ALT, AST, ALP, bilirubin, HDL	(47)
*F. sinkiangensis* PEA, NB, and M, fraction	50 µL	*DPPH assay* in *in vivo* study on HCT116, Caco-2, HepG2, and MFC cells	Free radical-scavenging activities, increased apoptosis, inhibited proliferation	(55)
*F. heuffelii* essential oil	22.43 µl/ml	DPPH radical scavenging assay	Reduced radical scavenging activity	(62)
*F. tingitana*	20 mg/ml	Breast (MCF7), cervical (HELA), and liver (HEPG2) carcinoma cell lines	Marked cytotoxic effects	(63)
*F. vesceritensis* essential oil	100 - 1000 mg/l	DPPH radical scavenging assay	Reduced radical scavenging activity	(61)
*F. assa-foetida* Oleo Gum Resin	5 × 10^−8^ to 10^−7^ g/ml	*In vitro* cultured human dermal fibroblasts (hdfs)	Reduced *β*-galactosidase activity, BCL2, p21, BAX, BAD, CASP3, and ALOX5	(26)
*F. szowitsiana* methanolic extract	10, 40 and 160 μg/ml	On human PHA-stimulated T lymphocytes	Decreased MDA, ROS, NO levels, increased CAT, SOD, and GSH	(28)
*F. assafoetida* essential oil	0.2-3.2 mg/ml	DPPH radical scavenging assay	Decreased nitric oxide-scavenging activity, Fe^2^ chelating ability, peroxidation inhibition	(48)
*F. * *assafoetida*	300, 400, and 500 µg/200 µl acetone/animal	TPA-mediated cutaneous oxidative stress in Swiss albino mice	Reduced hydrogen peroxide, xanthine oxidase activity, and PC	(49)
*F. foetida *hydroalcoholic E.	200-800 mg/kg, orally	Dexamethasone-induced hypertension in rats	Increased ferric reducing antioxidant power	(51)
*F. foetida*	68 and 352 mg/orally	Gentamicin-induced renal impairment in rat	Reduced BUN, Cr, and TBARS	(51)
*F. assafoetida*	1.25 and 2.5% w/w in diet	N-methyl-N-nitrosourea (MNU)-induced mammary carcinogenesis in rat	Increased activity of GSH-ST, DT-diaphorase, SOD, and CAT, decreased GSH level, inhibited lipid peroxidation	(52)
*F. assafoetida* hydroalcoholic E.	6.25 and 12.5 mg / Kg b.w	DMH-induced colon carcinogenesis in Wistar rats	Decreased cytochrome P_450_, β-catenin, ferric reducing ability, increased GSH and GSH S-T	(53)
*F. assafoetida*	0, 0.5, 1, and 2% in diet	Common carp	Increased GSR and GSTA, growth genes (GH, IGF1, and Ghrl)	(54)
*F. gummosa* root hydro-alcoholic E.	100 and 600 mg/kg /orally	Oro-gastric gavage in Wistar rats	Increased CAT and SOD activity, decreased serum MDA level	(56)
*F. communis*	150 and 300 mg/kg	CCl_4_-induced oxidative damage in rats	Decreased AST, ALT, GGT, and T-BIL serum levels but increased SOD and GPx activities	(57)
*F. pseudalliacea*	EC_50_, 65.29 ± 5.6 μM	Colorectal cancer cell lines (HCT116 and CT26)	DPPH radical scavenging	(58)
*F. * *assafoetida* E.	50 and 100 mg, daily	Children with leukemia	Reduced FBS, TC, TG, LDL, and MDA, increased HDL, SOD, CAT, and total antioxidant capacity	(65)

STZ: Streptozotocin, FBS: fasting blood sugar, TG: triglyceride, TC: total cholesterol, LDL: low density lipoprotein, HbA1c: , HDL: high density lipoprotein, MDA: malondialdehyde, SOD: superoxide dismutase, CAT: catalase, GSH: glutathione, GPx: glutathione peroxidase, GSH-ST: glutathione S-transferase, ALT: alanine transaminase, AST: aspartate transaminase, ALP: alkaline phosphatase, BUN: blood urea nitrogen, Cr: creatinine, GGT: γ-glutamyl transferase, T-BIL: total bilirubin, GH: growth hormone, IGF1: insulin growth factor 1, ROS: Reactive oxygen species, NO: nitric oxide, BCL2: B-cell lymphoma 2, p21: cyclin-dependent kinase inhibitor 1, BAX: Bcl2-associated X protein, BAD: BCL_2_ associated agonist of cell death (BAD) protein, CASP3: Caspase 3, ALOX5: Arachidonate 5-Lipoxygenase, PEA: petroleum ether, ethyl acetate, NB: n-butanol, TBARS: thiobarbituric acid reacting substances, DMH: 1, 2-dimethyl hydrazine, CCl4: tetrachloride, l-NAME: Nω-nitro-l-arginine methyl ester, E: extract, M: methanol, CAT: catalase, DPPH: 2,2-diphenyl-1-picrylhydrazyl, TPA: 12-O-Tetradecanoylphorbol-13-acetate, HCT116: human colon cancer cell line , Caco-2: human colorectal adenocarcinoma cells , HepG2: human liver cancer cell line. , and MFC cells: Microbial fuel cell, CT26: Animal fibroblast cells

**Table 4:** Anti-oxidant effects of the constituents of *Ferula* species

Constituents	Doses	Model of study	Effects	Ref.
Kamolonol acetate	2.5, 5, 10, 20, 40, and 80 μM	DPPH radical scavenging assay	Radical scavenging activity	(59)
Umbelliprenin	0.01 mmol/kg	Carrageenan-induced rat paw edema	Inhibited lipoxygenase activity	(33)
Kamolonol acetate	2.5, 5, 10, 20, 40, and 80 μM	HCT116, CT26, Vero and MSCs, DPPH antioxidant	Reduced radical scavenging activity	(58)
Auraptene	5, 10, 25, 50, 100, 200, and 400 mM	DNA damage in human T-cells	Antigenotoxic effects on DNA damage, reduced H_2_O_2_ genotoxicity	(66)
Auraptene	12.5, 25, and 50 mg/kg	Brain tissue of Kindling mice	Increased GSH levels	(67)
Auraptene	4, 8, and 25 mg/kg, orally	Vascular dementia and chronic cerebral hypoperfusion models	Decreased MDA but increased GSH	(68)
Umbelliprenin	10, 25, 50, 100, 200, and 400 μM	Human lymphocytes DNA lesions	Reduced DNA damage	(53)

GSH: glutathione, MDA: malondialdehyde, ROS: reactive oxygen species, MCF-7: Michigan Cancer Foundation-7, DPPH: 2,2-diphenyl-1-picrylhydrazyl, HCT116: human colon cancer cell line, H_2_O_2_: Hydrogen peroxide, DPPH: 2,2-diphenyl-1-picrylhydrazyl, MSCs: Mesenchymal stem cells, CT26: Animal fibroblast cells

**Table 5:** Immunomodulatory effects of the extract and constituents of *Ferula* species

Extract	Doses	Model of study	Effects	Ref.
Auraptene	0-20 𝜇g/ml	Jurkat T cells	Activation of caspase-8	(6)
Auraptene	0.5–15 mM	MTT colorimetric assay on splenocytes	Induced IL-4 level but decreased IFN- γ, NO, and PGE2 and inducible iNOS and COX	(32)
*F. assafoetida*	0, 0.5, 1, and 2% in diet	In carp	Increased TNF-α, IL-1β, IL-8, and LYZ, increased GH, IGF1, and Ghrl growth factors	(54)
Sesquiterpene chromone	30 µg/ml	Murine macrophage-like cell line (RAW 264.7)	Inhibited NO production and iNOS gene expression	(72)
Auraptene	10, 20, and 40 μM		Reduced CD3/CD28 cytokines and Th2 cytokine IL-4	(75)
Auraptene		Human HB4C5 cells hybridoma, mouse splenocytes, and mesenteric lymphocytes, *in vitro*	Increased IgM, IgA, and IgG.	(76)
Auraptene	40 and 200 mg/kg	Concanavalin A-activated splenocytes, *in vivo*	Increased immunoglobulin, IL-4, IFN-γ, and TNF-α production and B cell population	(76)
Auraptene	10, 30, and 90 μM	PHA-stimulated lymphocytes	Reduced lymphocytes proliferation, IL-4, IL-10, IFN-γ, NF-kB l, and NO levels, increased IFN-γ/IL-4 and IL-10/IL-4 ratio	(77)
Auraptene	250 μM	LPS -stimulated RAW 264.7 cells	Inhibited the production of PGE2, decreased COX-2 protein	(78)
Methyl galbanate	10 μM	LPS and IFN-γ -stimulated RAW264.7 mouse macrophage cells	Reduced NO production, iNOS mRNA, and COX-2 mRNA expression	(79)

MDA: Malondialdehyde, IFN-γ: interferon-gamma, IL-4: interleukin 4, NF-kB: nuclear factor-kB, NO: nitric oxide, Th2: T-helper2, MCF-7: Michigan Cancer Foundation-7, iNOS: inducible nitric oxide synthase, LYZ: lysozyme, PGE2: prostaglandin E2, IgM: immunoglobulin M, IgA: immunoglobulin A, IgG: immunoglobulin G, COX-2: cyclooxygenase-2, Bcl-2: associated X protein, TNF-α: tumor necrosis factor-alpha, LPS: Lipopolysaccharide, IFN-γ: interferon- γ, PHA: phytohemagglutinin, GH: grows factor, IGF1: insulin grows factor1, and Ghrl: Ghrelin and obestatin prepropeptide, SNU-1: Human cells, GLUT1: Glucose transporter 1, HK2: Hexokinase 2, PFK: Phosphofructokinase, PC3: human prostate cancer cell line, DU145: human prostate cancer cell line, CD44: cell-surface glycoprotein, LDHA: Lactate dehydrogenase A, Mcl-1: Induced myeloid leukemia cell differentiation protein

## Conclusion

Based on our literature review. The *Ferula* plants and their derivatives decrease the levels of inflammatory mediators and show anti-apoptotic effects. These plants and their constituents also decreased oxidative markers such as MDA, ROS, and NO but increased SOD, GPx, CAT, and GSH activities in various oxidative stress conditions. The immunomodulatory effects of *Ferula* plants and their compositions were also shown by their effects on various cytokines. Figure 3, summarized anti-inflammatory, anti-oxidant, and immunomodulatory effects of *Ferula* plants.

Therefore, the *Ferula* plants and their active constituents (UMB, MG, and terpenoid coumarins) could be potentially used as therapeutic targets for the treatment of a wide range of inflammatory, oxidative, and immune-dysregulatory disorders. In fact, while these plants are used in traditional medicine in many parts of the world, few clinical studies have assessed their therapeutic and biological effects. Importantly, these compounds’ safety is acceptable and they induce few side effects. However, further clinical studies are needed to scientifically document their therapeutic values. 

## Authors’ Contributions

ZG, MRA, and RR prepared the draft of the manuscript, MHB helped in the draft version and prepared its final version.

## Conflicts of Interest

The authors declare no conflicts of interest.
